# Orobol, A Derivative of Genistein, Inhibits Heat-Killed *Propionibacterium acnes*-Induced Inflammation in HaCaT Keratinocytes

**DOI:** 10.4014/jmb.2003.03063

**Published:** 2020-06-22

**Authors:** Yunsil Oh, Hwan Ju Hwang, Hee Yang, Jong Hun Kim, Jung Han Yoon Park, Jong-Eun Kim, Ki Won Lee

**Affiliations:** 1 Biomodulation Major and Research Institute of Agriculture and Life Sciences, Department of Agricultural Biotechnology, Seoul National University, Seoul 08826, Republic of Korea; 2 Center for Food and Bioconvergence, Seoul National University, Seoul 08826, Republic of Korea; 3 Department of Food Science and Biotechnology, Sungshin University, Seoul 01133, Republic of Korea; 4 Department of Food Science and Technology, Korea National University of Transportation, Jeungpyeong 27909, Republic of Korea

**Keywords:** Orobol, acne vulgaris, interleukin, inflammation, hyperkeratinization

## Abstract

Acne is a chronic skin disease that typically occurs in the teens and twenties, and its symptoms vary according to age, sex, diet, and lifestyle. The condition is characterized by hyperproliferation of keratinocytes in the epidermis, sebum overproduction, excessive growth of *Propionibacterium acnes*, and *P. acnes*-induced skin inflammation. Interleukin (IL)-1α and IL-6 are predominant in the inflammatory lesions of acne vulgaris. These cytokines induce an inflammatory reaction in the skin in the presence of pathogens or stresses. Moreover, IL-1α accelerates the production of keratin 16, which is typically expressed in wounded or aberrant skin, leading to abnormalities in architecture and hyperkeratinization. Orobol (3’,4’,5,7-tetrahydroxyisoflavone) is a metabolite of genistein that inhibited the *P. acnes*-induced increases in IL-6 and IL-1α levels in human keratinocytes (HaCaTs) more effectively compared with salicylic acid. In addition, orobol decreased the IL-1α and IL-6 mRNA levels and inhibited the phosphorylation of inhibitor of kappa-B kinase, nuclear factor of kappa light polypeptide gene enhancer in B-cells inhibitor, alpha, and mitogen-activated protein kinase induced by *P. acnes*. Finally, the expression of Ki67 was decreased by orobol. Thus, orobol ameliorated the inflammation and hyperkeratinization induced by heat-killed *P. acnes* and thus has potential for use in functional foods and cosmetics.

## Introduction

Acne is a chronic skin disease related to sebum and dead skin cells [1]. Usually beginning when hormone levels change during adolescence, acne occurs most commonly on the face, which has a high density of sebaceous glands, but also occurs at other sites, such as the neck, back, and chest [2]. In many cases, acne disappears upon reaching adulthood, although in some individuals, it lasts into adulthood [3]. In severe cases, the skin can become scarred. Severe acne can also cause psychological problems for young people who are sensitive about their appearance [4]. Acne is caused by a variety of factors and manifests with various clinical symptoms. Follicular hyperkeratinization, overgrowth of *Propionibacterium acnes*, inflammatory responses, and other genetic and environmental factors are involved [1]. Interleukin (IL)-1α and IL-6 are predominant in the inflammatory lesions of acne vulgaris. When the skin is affected by pathogens or damaged by stresses, these cytokines promote an inflammatory reaction [5]. IL-6 is a proinflammatory cytokine that mediates the immune response after stimulation by pathogen-associated molecular patterns. IL-1α accelerates the production of keratin 16, which is expressed in wounded or aberrant skin, leading to abnormalities in architecture and hyperkeratinization [2]. The keratinocytes in damaged epidermis express IL-1α [6], which stimulates the proliferation of neighboring cells and causes the skin to thicken. This may lead to hyperproliferation of keratinocytes and contribute to hyperkeratinization, aggravating acne [7, 8]. Therefore, inhibition of *P. acnes-*induced IL-6 and IL-1α expression could relieve the symptoms of acne [9].

**Fig. 1:**
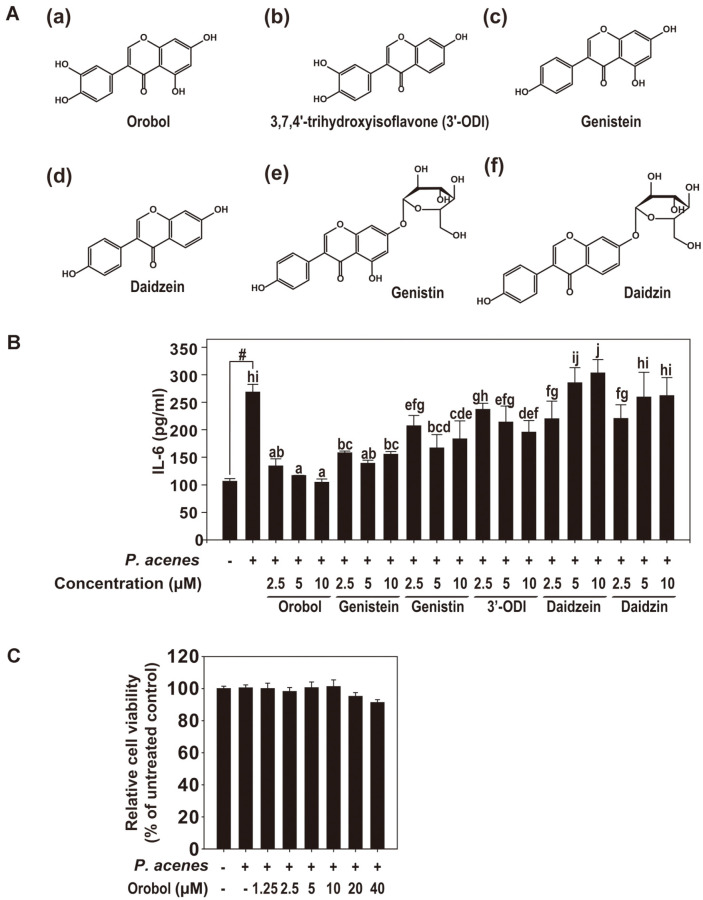
Anti-inflammatory effect of orobol in HaCaT cells. (**A**) Molecular structures of six isoflavones: (a) orobol, (b) 3,7,4’-trihydorxyisoflavone (3’-ODI), (c) genistein, (d) daidzein, (e) genistin, and (f) daidzin. *B*, Orobol inhibition of the *P. acnes*-induced upregulation of IL-6 in HaCaT cells. HaCaT cells were seeded into 96-well plates, cultured to 70–80% confluence in DMEM supplemented with 10% FBS, and starved in serum-free DMEM for 24 h. The cells were treated with the indicated concentrations of the isoflavones (**B**) for 1 h and treated with *P. acnes* [100 multiplicity of infection (MOI)] for 24 h. (**C**) Effect of orobol on the viability of HaCaT cells by MTT assay. HaCaT cells were treated with up to 40 μM orobol for 1 h and treated with *P. acnes* (100 MOI) for 24 h. #, significant difference between the control and *P. acnes*-treated groups (*p* < 0.05). One-way ANOVA with Duncan’s multiple-range test. Different letters indicate a significant difference at *p* < 0.05.

Isoflavone is a flavonoid of soybean that is known as a phytoestrogen because it has a similar structure and activity to estrogen [10]. The isoflavone content of soy is ~0.1–0.4%. The soy isoflavones comprise genistein, daidzein, glycitein, and glycosides (Fig. 1A). The effect of isoflavones on health has been investigated [10]. Isoflavones are widely used in foods and cosmetics to alleviate the effects of skin aging and inflammation while helping with moisturizing and whitening [11]. Genistein is the most abundant isoflavone in legume plants and has a variety of effects on health, including improved skin health. When genistein enters the body and is absorbed, various derivatives are produced [12], such as orobol, a rare isoflavone. We previously developed an enzyme-based method for mass production of orobol and reported its anti-obesity effects [13]. In this study, orobol decreased the *P. acnes-*induced production of IL-1α and IL-6 in human keratinocytes (HaCaT cell line) in a dose-dependent manner. Orobol also decreased IL-1α and IL-6 mRNA levels; inhibited the phosphorylation of inhibitor of kappa-B kinase (IKK), nuclear factor of kappa light polypeptide gene enhancer in B-cells inhibitor, alpha (IκBα), and mitogen-activated protein kinase (MAPK); and decreased the expression of Ki67, induced by heat-killed *P. acnes*. Orobol inhibits the proliferation of *P. acnes* and thus could reduce the inflammation and hyperkeratinization induced by *P. acnes*.

## Materials and Methods

### Chemicals and Reagents

Orobol was provided by Prof. Byung-Gee Kim (Seoul National University, Korea). Orobol was o-hydroxylated by genistein with a > 99.9% conversion yield. It underwent biotransformation by tyrosinase expressed in *Bacillus megaterium*. Genistein was purchased from Sigma-Aldrich (USA). Dulbecco’s modified Eagle’s medium (DMEM) was purchased from Wel-Gene (Republic of Korea). Fetal bovine serum (FBS) was purchased from Sigma-Aldrich and penicillin/streptomycin solution from Mediatech, Inc. (USA). Antibodies against phosphorylated IKKα/β, total IKKα, total IKKβ, phosphorylated IκBα, total IκBα, phosphorylated mitogen- activated protein kinase kinase (MEK)1/2 (Ser217/Ser332), total MEK1/2, phosphorylated dual-specificity mitogen- activated protein kinase kinase (MKK)3/6 (Ser189/Ser207), total MKK3β, phosphorylated MKK4, total MKK4, phosphorylated p38, total p38, phosphorylated c-Jun N-terminal kinase (JNK), total JNK, phosphorylated p90 ribosomal s6 kinase (RSK), total RSK, phosphorylated mitogen- and stress-activated protein kinase 1 (MSK1) (Ser360), and total MSK1 were purchased from Cell Signaling Technology Inc. (USA). Antibodies against phosphorylated extracellular signal-regulated protein kinases 1 and 2 (ERK1/2) (Thr202/Tyr204), total ERK1/2, and β-actin were purchased from Santa Cruz Biotechnology (USA). Protein assay kits were obtained from Bio- Rad Laboratories (USA).

### Cell Culture and Treatment

HaCaT cells were purchased from Cell Lines Services GmbH (Germany). HaCaT cells were cultured in Dulbecco’s modified Eagle’s medium (DMEM) containing 10% (v/v) FBS and 1% (v/v) penicillin/streptomycin at 37°C in a 5% CO_2_ atmosphere.

### Preparation of *P. acnes*

*P. acnes* was cultured on agar under anaerobic conditions by the spreading method for 4–5 days. Colonies were harvested using an inoculating loop and dissolved in 50 ml agar broth. Next, the absorbance of the tubes was assayed using a spectrophotometer, and the tubes were centrifuged at 10,000 g and 4°C for 10 min. The supernatants were discarded, and the pellets were washed in 1X phosphate-buffered saline (PBS). After two further centrifugation and washing steps, the tubes were placed in a preheated stirrer at 65°C for 30 min and centrifuged at 10,000 g and 4°C for 10 min. The supernatants were discarded, and the pellets were resuspended in serum-free DMEM. The samples were vortex mixed, aliquoted into 2.0 ml tubes, and stored at −80°C.

### Cell Viability Assay

Cytotoxicity was measured by 3-(4,5-dimethylthiazol-2-yl)-2,5-diphenyltetrazolium bromide (MTT) assay. HaCaT cells were cultured in a 96-well plate at 2 × 10^4^/well in DMEM containing 10% FBS and penicillin/ streptomycin at 37°C in a 5% CO_2_ atmosphere. HaCaT cells were starved in serum-free DMEM for 12 h, treated with the samples (100 μl/well), and incubated for 23 h. Next, MTT solution was added to the cells for 1 h. The resulting formazan product was dissolved in DMSO, and the absorbance at 480 nm was measured using a microplate reader (Molecular Devices, USA).

### Luciferase Reporter Assay

HaCaT cells transfected with the luciferase gene at the nuclear factor-kappa-B (NF-κB) and AP-1 positions were cultured for 24 h in 96-well plates at 2.0 × 10^4^/well. The cells were next incubated in serum-free DMEM for 12 h and pretreated with orobol in serum-free DMEM for 1 h and then with *P. acnes* [multiplicity of infection (MOI), 100] for 12 h. The cells were washed twice with 200 μl 1X PBS, and the plasma and nuclear membranes were lysed by adding 80 μl lysis buffer with agitation. The samples were transferred to a non-transparent, white 96-well plate. Luciferase activity was determined using a microplate reader (Promega, USA) and Glowmax software.

### Enzyme-Linked Immunosorbent Assay

HaCaT cells were seeded in a 12-well plate at 2.5 × 10^5^/well, incubated for 24 h, and subsequently incubated in serum-free DMEM for 12 h. Next, the cells were pretreated with orobol for 1 h, followed by heat-killed *P. acnes* (MOI, 100) for 24 h. The cultures were harvested by centrifugation at 4°C for 10 min; 80% of the supernatant was collected and assayed. Immunoplates were purchased from SPL Life Sciences Inc. (Republic of Korea). Antibodies, reagents, and substrates for IL-1α and IL-6 were purchased from R&D Systems, Inc. (USA). Enzyme- linked immunosorbent assays (ELISAs) were performed according to the manufacturer’s instructions. The absorbance was measured using a microplate reader.

### Real-Time Quantitative PCR

HaCaT cells were seeded in a six-well plate at 5.0 × 10^5^/well, incubated for 24 h, and then incubated in serum- free DMEM for 12 h. The cells were treated with orobol and genistein for 1 h followed by heat-killed *P. acnes* (MOI, 100) for 1 h. RNAiso Plus (TaKaRa Bio Inc., Japan) was used to extract mRNA. The mRNA was dissolved in DEPC water and quantified using the NanoDrop ND-2000 spectrophotometer (Thermo Fisher Scientific, USA). Reverse transcription using oligo-dT primers was performed using the PrimeScript One-Strand cDNA Synthesis Kit (TaKaRa Bio, Inc.). IQ SYBR mixture was added to the primers and cDNA in a 96-well PCR plate in triplicate. β- actin was used as the loading control. The primer sequences were as follows: IL-1α forward (5′- TGGTAGTAGCAACCAACGGGA-3′) and reverse (5′-ACTTTGATTGAGGGCGTCATTC-3′); IL-6 forward (5′-CAATCTGGATTCAATGAGGAGAC-3′) and reverse (5′-CTCTGGCTTGTTCCTCACTACTC-3′); and β- actin forward (5′-TCCTCACCCTGAAGTACCCCAT-3′) and reverse (5′-AGCCACACGCAGCTCATTGTA-3′).

### Determination of Minimum Inhibitory Concentration

The minimum inhibitory concentrations (MIC) were determined by the Culture Collection of Antimicrobial Resistant Microbes (CCARM, Republic of Korea) according to the guidelines of the Clinical Laboratory Standards Institute. The MICs against *Escherichia coli* CCARM 0012 and *Staphylococcus aureus* CCARM 3102 were determined by plate culture dilution using Muller Hinton I medium (BBL, USA) with norfloxacin as the control antibiotic. *E. coli* ATCC 25922 and *S. aureus* ATCC 29213 were used as control strains. The MIC against *P. acnes* CCARM 9008 was determined by the microfluidic dilution method in cation-adjusted Muller Hinton II broth with 5% lysed horse blood (BBL). Clindamycin was used as a control antibiotic. *Streptococcus pneumoniae* CCARM 0031 was used as a control strain.

### Western Blotting

HaCaT cells were seeded in 6 cm dishes at 5.0 × 10^5^/well for 24 h and incubated in serum-free DMEM for 12 h. Next, the cells were treated with orobol for 1 h followed by heat-killed *P. acnes* (MOI, 100). Cytosolic and nuclear proteins were dissolved in radioimmunoprecipitation assay buffer, left on ice, vortex mixed three times for 10 min each, and centrifuged at 14,000 g and 4°C for 10 min. The supernatants were subjected to protein quantification and western blotting. Proteins were resolved by 10% sodium dodecyl sulfate–polyacrylamide gel electrophoresis and transferred to polyvinylidene difluoride membranes (Millipore, USA). The membranes were blocked in 5% skim milk for 1 h and incubated with the primary antibodies at 4°C on a linear shaker overnight. After washing in Tris-buffered saline with Tween, the membranes were incubated with horseradish peroxidase-conjugated secondary antibodies (Santa Cruz Biotechnology) diluted 1:5,000 in skim milk, at room temperature on a linear shaker for 1 h. Protein bands were visualized using enhanced chemiluminescence detection reagents (Amersham, GE Healthcare Life Sciences, UK). Band intensities were quantified using ImageJ (National Institutes of Health, USA).

### Immunofluorescence

Coverslips were coated with poly-D-lysine for at least 4 h and with collagen (type 1) under UV light. HaCaT cells were seeded onto the coverslips in a 3 cm dish at a density of 5.0 × 10^5^/dish and incubated for 24 h. After each step, the cells were washed with 1X PBS and fixed with 4% formaldehyde. Before blocking, 0.1% Triton-X in 1X PBS was added as a permeation agent. After blocking, primary antibodies in blocking solution were added to the samples overnight at 4°C. Next, fluorescein-conjugated secondary antibodies were added to the samples for 2 h at room temperature in darkness. For counterstaining, 4′,6-diamidino-2-phenylindole (DAPI) was used. Finally, the coverslips were placed over mounting medium and visualized under a confocal microscope (Zeiss LSM 880, Carl Zeiss, Germany).

### Statistical Analysis

Results are presented as means ± standard deviation (SD). The significance of the differences was assessed by Student’s *t*-test or one-way analysis of variance (ANOVA) with post hoc Duncan’s test, as appropriate. Image analysis was performed using ImageJ and the statistical analysis using IBM SPSS Statistics ver. 22.0 (IBM Co., USA). A *p*-value < 0.05 was taken to indicate statistical significance.

## Results

### Orobol Significantly Reduces the *P. acnes*-Induced Increase in the IL-6 Level in HaCaT Cells

The induction of cytokine synthesis by *P. acnes* plays an important role in the development of skin inflammation. IL-6, one of the best-characterized proinflammatory cytokines, is expressed in keratinocytes under a variety of conditions [9]. We tested the effects of six isoflavones (Fig. 1A), including genistein and daidzein, the most abundant isoflavones in soybean, on keratinocytes. Among the six isoflavones, 10 μM orobol inhibited the *P. acnes*-induced increase in the IL-6 level most effectively (Fig. 1B) without exerting a cytotoxic effect (Fig. 1C).

### Orobol Inhibits the Proliferation of *P. acnes*

Substances with antimicrobial activity against *P. acnes* can alleviate the symptoms of acne [14]. We thus tested the antibacterial effect of orobol on *P. acnes* by the agar-diffusion test and by determining the MIC. In the agar-diffusion test, 1 mg orobol resulted in a zone of 21 mm diameter, compared with 18 mm for the quality-control sample. The MIC of orobol for *P. acnes* was 0.313 mM/ml, suggesting that orobol inhibits the proliferation of *P. acnes*.

### Orobol Inhibits the *P. acnes*-Induced IL-6 and IL-1α Expression in, and the IL-1α-Induced Proliferation of, HaCaT Cells

**Fig. 2:**
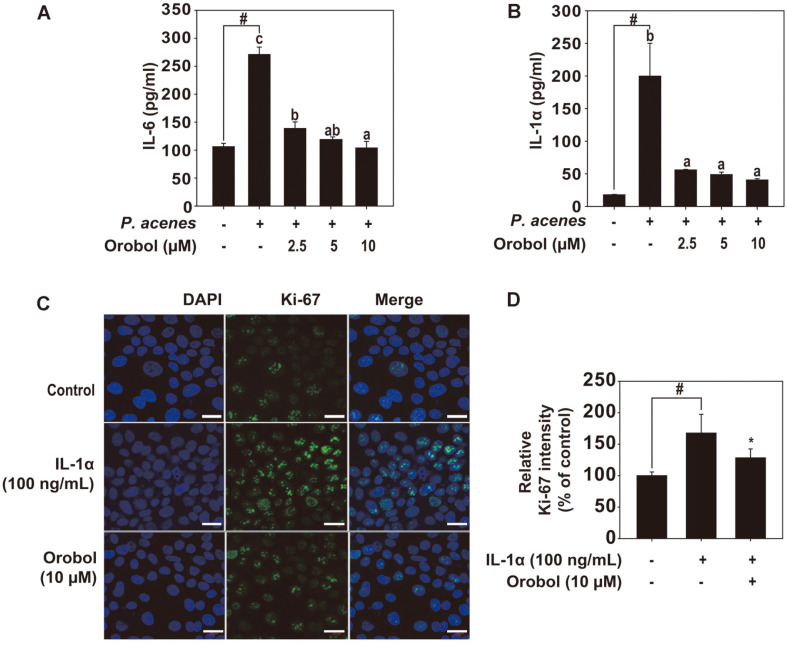
Effect of orobol on acne. (**A** and **B**) Orobol inhibition of *P. acnes*-induced IL-6 and IL-1α expression in HaCaT cells. HaCaT cells were seeded into 96-well plates, cultured to 70–80% confluence in DMEM supplemented with 10% FBS, and starved in serum-free DMEM for 24 h. The cells were treated with the indicated concentrations of orobol for 1 h and with *P. acnes* (100 MOI) for 24 h. IL-6 (**A**) and IL-1α (**B**) levels in conditioned medium, measured by ELISA. (**C** and **D**) Effect of orobol on HaCaT proliferation. HaCaT cells were pretreated with 10 μM orobol for 1 h and stimulated with 100 ng/ml human IL-1α. Cell proliferation was assayed by measuring the intensity of Ki67 fluorescence. Cells were counterstained with DAPI. (**D**) Relative intensities of Ki67 in the control and induction groups. #, significant difference between the control and *P. acnes*-treated groups (*p* < 0.05). One-way ANOVA with Duncan’s multiple-range test. Different letters indicate a significant difference at *p* < 0.05.

The *P. acnes*-induced production of IL-6 and IL-1α plays an important role in skin inflammation. *P. acnes* significantly increased the IL-6 and IL-1α levels in HaCaT cells (Figs. 2A and 2B). This suggests that orobol can ameliorate *P. acnes*-induced skin inflammation. IL-1α is synthesized by keratinocytes and promotes their proliferation by binding to the IL-1 receptor. To determine whether orobol reduced hyperproliferation of keratinocytes, we assayed Ki67 expression by immunofluorescence (Figs. 2C and 2D). Orobol-treated keratinocytes showed significantly decreased Ki67 expression compared with those treated with IL-1α. Orobol also decreased the hyperproliferation of HaCaT keratinocytes induced by IL-1α. Therefore, orobol reversed the cytokine production induced by *P. acnes* in keratinocytes and reduced their hyperkeratinization.

### Orobol Decreases the mRNA Levels of Proinflammatory Cytokines via NF-κB and AP-1 Transactivation

**Fig. 3:**
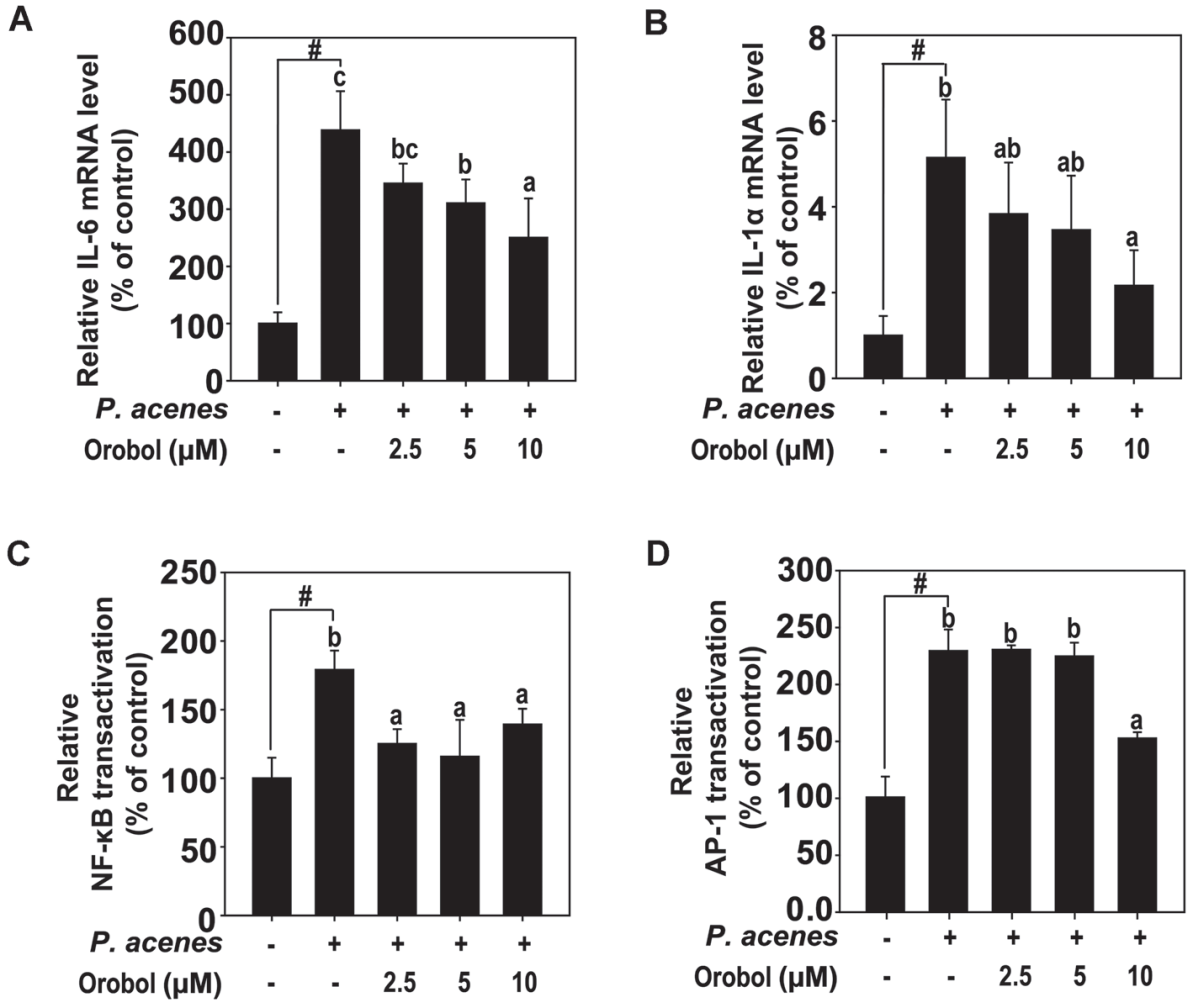
Effect of orobol on *P. acnes-*induced IL-6 and IL-1α expression and NF-κB and AP-1 transactivation. (**A** and **B**) IL-6 and IL-1α mRNA levels measured by RT-qPCR. HaCaT cells were pretreated with orobol for 1 h and treated with *P. acnes* (100 MOI) for 24 h. **(C** and **D**) Orobol suppression of *P. acnes*-induced NF-κB (**C**) and AP-1 (**D**) transactivation in HaCaT cells stably transfected with NF-κB or AP-1 luciferase reporter plasmids. Relative luciferase activities are presented as means ± SD. #, significant difference between the control and *P. acnes*-treated groups (*p* < 0.05). One-way ANOVA with Duncan’s multiple range test. Different letters indicate a significant difference at *p* < 0.05.

*P. acnes* significantly increased the mRNA levels of IL-6 (Fig. 3A) and IL-1α (Fig. 3B) in HaCaT cells, and orobol inhibited these increases. Furthermore, orobol decreased the activities of NF-κB (Fig. 3C) and AP-1 (Fig. 3D).

### Orobol Decreases the *P. acnes*-Induced Phosphorylation of MAPKs and NF-κB

**Fig. 4:**
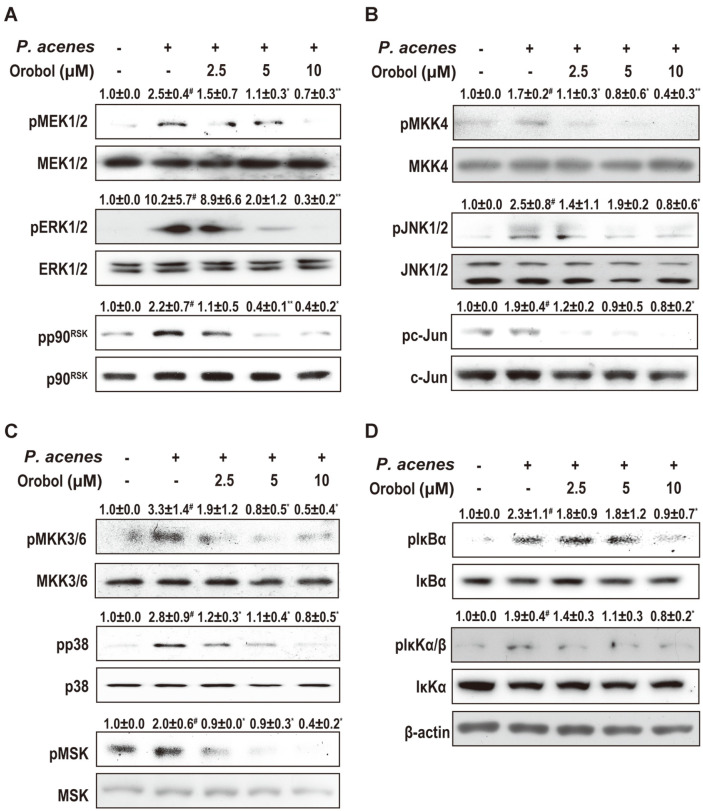
Effect of orobol on *P. acnes*-induced signaling in HaCaT cells. HaCaT cells were treated with orobol (0, 2.5, 5, or 10 μM) for 1 h, treated with *P. acnes* (100 MOI) for 15 min, and subjected to western blotting. Protein band densities were measured using ImageJ and normalized to that of β-actin. Data are means ± SD of at least three independent experiments. #, significant difference between the control and *P. acnes*-treated groups (*p* < 0.05). One-way ANOVA with Duncan’s multiple-range test. Different letters indicate a significant difference at *p* < 0.05.

The above results indicate that orobol ameliorates inflammation by inhibiting NF-κB and AP-1. To activate NF- κB and AP-1, upstream MAPKs must be phosphorylated. Thus, the effect of orobol on the phosphorylation of MAPKs in HaCaT cells was evaluated by western blotting. Orobol decreased the phosphorylated MEK1/2, ERK, and p90RSK levels compared with the levels induced by *P. acnes* (Fig. 4A). Likewise, orobol decreased the phosphorylated MKK4, JNK, and c-jun (Fig. 4B) and MKK3/6, p38, and MSK1 (Fig. 4C) levels compared with *P. acnes*. The activity of NF-κB is regulated by its phosphorylation by IKKα/β and IκBα. The levels of phosphorylated IKKα/β and IκBα were decreased by orobol (Fig. 4D). Therefore, orobol suppresses inflammation by inhibiting the activation of NF-κB and AP-1.

## Discussion

Orobol is a rare isoflavone produced in trace amounts during prolonged fermentation of soybean [15]. We discovered an enzyme that catalyzes the production of orobol from genistein and that can easily be extracted from soybeans [16, 17]. Orobol attenuates high-fat-diet-induced weight gain and lipid accumulation without affecting food intake in C57BL/6J mice, in addition to casein kinase 1 epsilon activity [13]. Orobol also has antiviral activity [18]. We report here that the anti-inflammatory activity of orobol mediates its beneficial effect on acne.

Acne is a common relapsing chronic skin disease related to sebum and dead skin cells [4], and its symptoms vary depending on age, sex, hormones, diet, and lifestyle. Sebaceous glands are found in many areas of the face, back, and chest, where acne is common [19]. The sebaceous glands produce sebum and are connected to tubes containing hair follicles [9]. Under normal conditions, sebum rises along the hair-follicle wall and is secreted through the skin. However, when the hair follicles become clogged, sebum is trapped in the sebaceous glands, promoting the proliferation of inflammation-causing bacteria [20]. Treatments for acne suppress the secretion of sebum and remove clogged hair follicles so that sebum is released [21]. The symptoms caused by *P. acnes* can be alleviated by preventing inflammation. In this study, orobol inhibited the *P. acnes*-induced increase in cytokine production and inhibited inflammation. Orobol has been reported to suppress the growth of *P. acnes* and the production of sebum [22]. Therefore, orobol has potential for alleviating the symptoms of acne.

*P. acnes* causes inflammation, exacerbating acne. IL-1α causes keratosis of the hair follicles and sebaceous glands, causing acne. IL-1α is highly expressed in sebaceous epithelial cells and hair follicles in patients with acne [6]. IL-1α promotes the aggregation of various adhesion molecules in vascular endothelial cells and promotes the attachment of white blood cells to the endothelium and their production of proinflammatory cytokines [6]. Orobol reduces the production of proinflammatory cytokines and inhibits *P. acnes*-induced skin inflammation. Orobol also prevents the hyperproliferation of keratinocytes induced by IL-1α. IL-6 exacerbates acne [23] by promoting inflammation in the skin. Orobol inhibits the *P. acnes*-induced expression of IL-6. Therefore, orobol may ameliorate the inflammation induced by *P. acnes*.

*P. acnes* activates Toll-like receptors (TLR)-2 and TLR-4 to induce the synthesis of various cytokines by activating NF-κB or AP-1, which are regulated by MAPK [21] and themselves regulate IL-6 and IL-1α expression. Orobol inhibited the *P. acnes*-induced expression of IL-6 and IL-1α as well the activities of NF-κB and AP-1. Phosphorylation of ERK1/2, JNK1/2, and p38 was induced by *P. acnes* and was inhibited by orobol. Orobol inhibited MAPK-induced NF-κB and AP-1 transactivation and reduced IL-6 and IL-1α mRNA and protein levels. The efficacy of an isoflavone is based on its activity as a phytoestrogen and regulation of phytoestrogen downstream signaling. Although orobol has a lower binding activity to estrogen receptors than do other isoflavones [24], it was the most efficacious compound in this study. This suggests that the effect of orobol is not mediated by its estrogenic activity. Further work should focus on identifying the molecular targets of orobol to determine the mechanism underlying its effect on the inflammation caused by acne.
